# Flea-borne *Rickettsia* species in fleas, Caldas department, Colombia

**DOI:** 10.3855/jidc.12524

**Published:** 2020-10-31

**Authors:** Carol B Colonia, Alejandro Ramírez-Hernández, Juliana Gil-Mora, Juan C Agudelo, Gabriel Jaime Castaño Villa, Camilo Pino, Paola Betancourt-Ruiz, Jorge E Pérez Cárdenas, Lucas S Blanton, Marylin Hidalgo

**Affiliations:** 1 Grupo de Enfermedades Infecciosas, Facultad de Ciencias, Pontificia Universidad Javeriana, Bogotá D.C., Colombia; 2 Grupo Parasitología Veterinaria, Facultad de Medicina Veterinaria y de Zootecnia, Universidad Nacional de Colombia, Bogotá D.C., Colombia; 3 Rickettsial and Ehrlichial Disease Research, Department of Pathology, University of Texas Medical Branch, Galveston, TX, United States; 4 Facultad de Ciencias Agropecuarias, Universidad de Caldas, Manizales, Caldas, Colombia; 5 Grupo de investigación GEBIOME, Departamento de Desarrollo Rural y Recursos Naturales, Facultad de Ciencias Agropecuarias, Universidad de Caldas, Manizales, Caldas, Colombia; 6 Laboratorio de Investigación en Sistemas Inteligentes, Facultad de Ingeniería, Universidad Nacional de Colombia, Bogotá D.C., Colombia; 7 Facultad de Ciencias para la Salud, Universidad de Caldas, Manizales, Caldas, Colombia; 8 Division of Infectious Diseases, Department of Internal Medicine, University of Texas Medical Branch, Galveston, TX, United States

**Keywords:** *Rickettsia felis*, *Rickettsia asembonensis*, RFLP, vector-borne diseases, zoonotic diseases

## Abstract

**Introduction:**

Rickettsioses are zoonotic diseases caused by pathogenic bacteria of the genus *Rickettsia* and transmitted to man by means of arthropod vectors such as ticks, fleas, mites and lice. Historically, Caldas Department has reported a significant number of cases of murine typhus to the Colombian national health surveillance system, and consequent studies of flea-borne rickettsiosis identified the circulation of *Rickettsia typhi* and *Rickettsia felis* in multiple municipalities. Our aim was to genotype species of *Rickettsia* detected in fleas collected from domestic and wild mammals in Caldas.

**Methodology:**

Flea samples were taken by convenience sampling from dogs, cats and wild mammals (rodents and marsupials) in 26 municipalities. Specimens were classified by current taxonomic keys and pooled for DNA extraction and molecular screening for *Rickettsia* spp. by PCR amplification of *gltA, htrA* and *sca5* genes. Positive samples were genotyped by enzyme digestion (*htrA*) and sequencing.

**Results:**

A total of 1388 flea samples were collected. *Rickettsia* DNA was amplified in 818 (*gltA*), 883 (*htrA*) and 424 (*sca5*) flea pools. Alignment analysis with available *Rickettsia* DNA sequences showed greater similarity with *R. asembonensis* (*gltA*) and with *R. felis* (*sca5* and *htrA*). Restriction pattern was compatible with *R. felis*. *R. typhi* was not identified.

**Conclusion:**

The present study confirms the presence and high prevalence of *R. asembonensis* and *R. felis* in fleas from domestic and wild animals in different municipalities from Caldas Department.

## Introduction

*Rickettsia* spp. are obligately intracellular bacteria belonging to the family Rickettsiaceae (order Rickettsiales), which can cause mild to severe diseases in humans and other animals [1]. Historically, two flea-borne rickettsial species have been recognized (i.e., *Rickettsia typhi* and *Rickettsia felis*) [2], but recently, new *R*. *felis*-related species (i.e., *Rickettsia asembonensis* sp. nov. and ‘*Candidatus* Rickettsia senegalensis’) and others have been described [3–5].

*Rickettsia typhi* is the etiologic agent of murine or endemic typhus, a febrile zoonotic disease which involves the Oriental rat flea (*Xenopsylla cheopis*) and different rodents (e.g., *Rattus* spp.) in its enzootic cycle [6]. Murine typhus has a wide distribution in tropical regions throughout the world and is currently recognized as endemic in parts of South America, Australia, Asia and southeastern Europe [7–11]. The disease is also endemic in California and Texas (USA), where an alternate suburban transmission cycle, apparently involving opossums and cat fleas (*Ctenocephalides felis*), has been described [12,13].

*Rickettsia felis*, a species discovered within a *C*. *felis* laboratory colony in 1990 [14], has been molecularly detected in a diversity of arthropods including other flea species, ticks, mites, booklice and even mosquitoes [15]; nonetheless, the cat flea is the only recognized vector and reservoir [2]. Reports of human infections have increased in recent years, but the role of *R. felis* as a cause of disease has been scrutinized [2,16,17].

Several studies have suggested that flea-borne *Rickettsia* species (*R. typhi* and *R. felis*) can share vectors such as the cat flea (*C. felis*) and the dog flea (*C. canis*); however, *R. felis* is the most frequently detected species [18,19]. Importantly, the role of cats, dogs and other animals as possible reservoirs of these *Rickettsia* species remains unknown [20–22].

In Colombia, Caldas Department has been considered an endemic area for murine typhus since 1942 [23]. Recent studies in this region have established a seroprevalence of 25.5% and 17.8% for *R*. *typhi* and *R. felis*, respectively, and 28.7% for both species [24]. Likewise, another report confirmed, by seroconversion, the diagnosis of 12 patients with signs and symptoms suggestive of murine typhus [25]. In 2013, the presence of *R. felis* in fleas collected from animals was reported, for the first time, in the same area in Colombia [26]. Despite this, the circulation of other flea-borne *Rickettsia* species in Caldas remains unknown.

The aim of this work was to detect and genotype rickettsial DNA from flea samples collected from domestic and wild animals in order to contribute to the knowledge of flea-borne *Rickettsia* species in this endemic region.

## Methodology

### Flea sampling

This cross-sectional study was conducted between November 2015 and January 2017 and was approved by the ethics committees of the Pontificia Universidad Javeriana and Universidad de Caldas (Act 8^th^, June 9^th^, 2014; and CBCS-016–14, May 28^th^, 2014, respectively).

The area of study was the Department of Caldas, located in the midwestern area of Colombia (central branch of the Andes). Sampling was performed in urban and rural zones of 26 municipalities as listed in Table 1; a map and geographic coordinates are presented as supplemental material (Supplementary Figure 1 and Supplementary Table 1).

Domestic animals (dogs and cats) and wild mammals (synanthropic and non-synanthropic species) were included in the study for ectoparasite sampling. Dogs and cats, in urban and rural households, were sampled after the owner’s consent and proper manual restraint. Wild mammals were captured by live trapping. For these, Sherman and Tomahawk traps were distributed in parallel transects of variable longitude and separated between them by 20 to 50 meters, depending on the local topography. Traps were placed 10 meters apart and at two alternate heights (ground-level and two-meter level). A mixture of banana, cereal, vanilla essence and sardines were used as bait during three nights of sampling with a total sampling effort of 2148 trap-nights. Captured animals were anaesthetized with isoflurane within a hermetic plastic box (3 to 5-minute exposure depending on body size) and submitted to morphometric and photographic measures for further taxonomic classification. All individuals were released to nature after recovery.

Fleas were collected manually or by hair combing from domestic dogs, cats and wild mammals. All specimens were stored in 70% ethanol, further classified by current morphological keys [27–29] and preserved at −20 °C prior to DNA extraction. For this, specimens were grouped into pools (2–3 fleas/pool), considering the following criteria: flea species, animal host and geographic origin.

### DNA extraction

Flea samples were submitted to a dry bath (56 °C for 30 minutes) to eliminate any ethanol trace and DNA was further extracted with a modified protocol using guanidine thiocyanate (DNAzol; InvitrogenTM, Life Technologies Corp., Grand Island, NY, USA) and the DNeasy Blood and Tissue Kit (Qiagen TM, Germantown, MD, USA), as previously reported [26]. Subsequently, DNA purity and concentration were measured using a calibrated spectrophotometer (West Tune NanoGenius series, Hangzhou, China).

### Rickettsia DNA amplification

Before performing PCR reactions for *Rickettsia*, we assessed the presence of amplifiable DNA and the absence of inhibitors in the extracted flea samples by amplification of cytochrome oxidase subunit II (*COII*) gene (primers COII-F-Leu and COII-F-Lys) [30].

The detection of *Rickettsia* DNA in flea samples was performed by the amplification of *gltA* (primers CS −78-CS323 and CS5-CS6) [31], *htrA* (17kD1–17kD2) [31] and *sca5* (also known as *ompB*) genes (120.M59–120.807 and 120.607 F-120.1497R) [32]. *Rickettsia slovaca* DNA was used as a positive control and water as a negative control in all reactions. For positive flea pools, Minimal Infection Rates (MIR) were calculated for each of the three genes evaluated, as previously reported [33].

### RFLP

Amplified *htrA* products were submitted to endonuclease digestion using the enzymes AluI and XbaI [13]. Further analysis of the restriction sites was carried out with the NEBcutter V2.0 program (New England Biolabs, Inc., Ipswich, MA, USA) in order to identify fragment patterns and genotype of the *Rickettsia* species.

### DNA Sequencing

With the aim to identify *Rickettsia* species circulating in fleas, some PCR positive samples were reamplified with a proof-reading *Taq* enzyme PCR protocol and purified with ExoSAP-IT Express PCR Cleanup kit (Thermo Fisher Scientific, Waltham, MA, USA). Thereafter, they were submitted for Sanger automatized capillary sequencing (ABI-3500XL Genetic Analyzer, Applied Biosystems, Waltham, MA, USA). Flea pools samples positive for the three genes evaluated were submitted for sequencing, excepting those from *Xenopsylla cheopis* which could be positive for at least one gene.

### Bioinformatic analysis

The pre-processing of the sequence data was performed using Trace Tuner [34] and CAP3 [35] programs. Nucleotide-nucleotide alignment analysis was performed using FASTA files with a rickettsial genome database obtained from NCBI assembly [36], through NCBI Taxonomy [37], including sequences from this study and those obtained in the work published by Ramírez-Hernández *et al*. [26]. The ClustalW program [38] was used for refining alignments prior to construction of phylogenetic trees. These were built using the Maximum Likelihood method based on the Tamura-Nei model [39]. Branch support was tested by bootstrap analysis using 1000 replicates. *Rickettsia canadensis* was used as an outgroup (accession numbers: MH595545.1 and CP000409.1). Trees were constructed and analyzed with MEGA 7 software [40].

## Results

### Rickettsia detection in flea samples

In total, 1388 fleas were collected in 23 out of 26 municipalities (none were collected in Aguadas, La Merced and Salamina). 1344 (96.8%) were from domestic animals (584; 43.5% from dogs and 760; 56.6% from cats) and 44 (3.2%) from wild mammals (Table 1). *C. felis* was the most prevalent species (1099; 79.4%) followed by *C. canis* (258; 18.6%). Other flea species included *Leptopsylla segnis* (10; 0.7%), *X*. *cheopis* (8; 0.6%), *Pulex irritans* (4; 0.3%) *Ctenophthalmus* sp. (4; 0.3%), *Nosopsyllus* sp. (2; 0.1%), *Leptopsylla* sp. (2, 0.1%) and *Rhopalopsyllus* sp. (1; 0.07%) (Table 1).

In total, 911 pools were grouped using the criteria mentioned. The cytochrome oxidase *(COII)* gene was amplified from all flea pools. Globally, 818 (89.8%), 424 (46.5%) and 883 (96.9%) flea pools were positive for *gltA*, *sca5* and *htrA* genes, respectively (Table 2). As presented in Table 2, total MIR ranged from 0.3 (*gltA*) to 0.7 (*htrA*) with values by municipalities that achieved up to 1.0 (i.e. 100% of infection in Chinchiná, Manizales, Marmato, Pensilvania, Samaná, Victoria and Villamaria).

Amplification of the three *Rickettsia* genes was achieved in 382 flea pools, which were considered for RFLP analysis. By species, 367 (96.1%) and 15 (3.9%) pools from *C*. *felis* and *C*. *canis*, respectively, were positive for the three genes. In contrast, none of the other flea species amplified all genes.

### Rickettsia species identification by RFLP in flea samples

**Figure 1.**
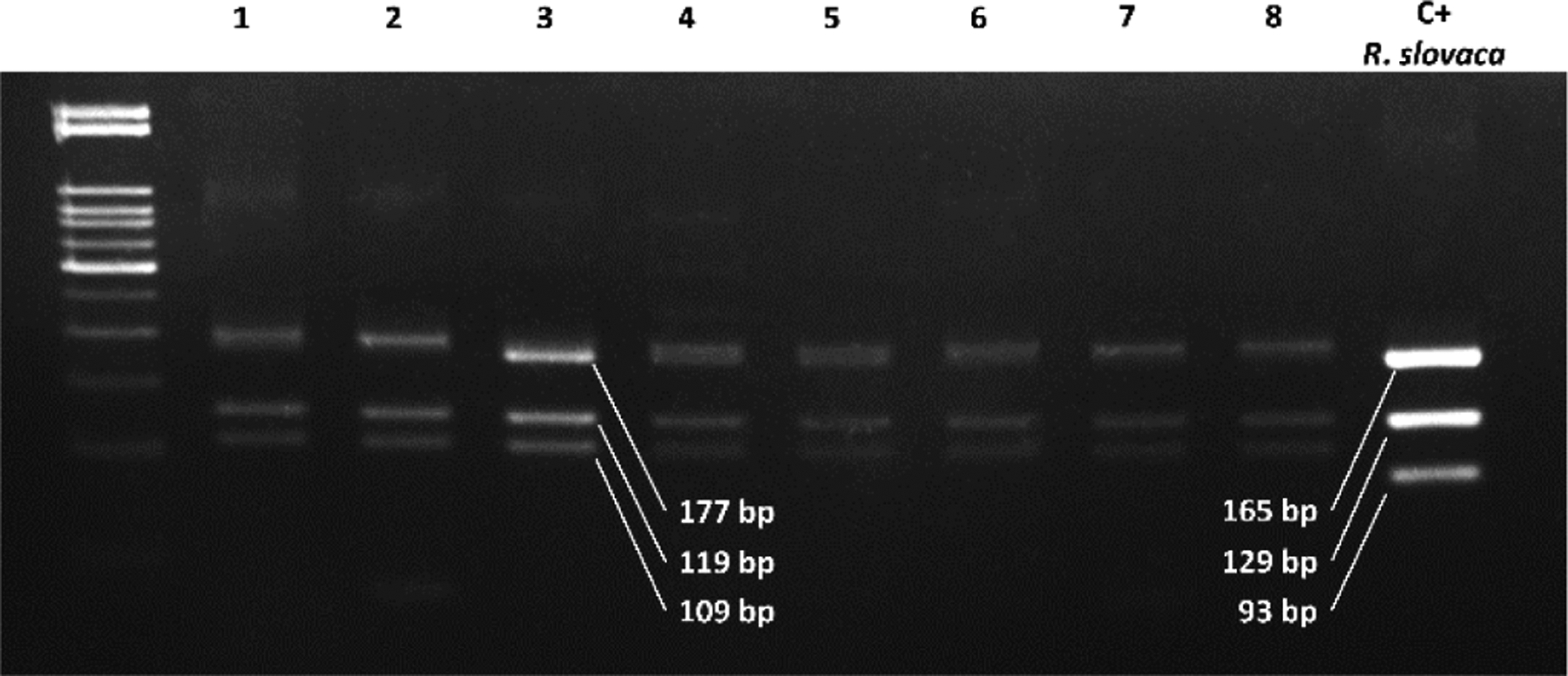
Restriction patterns with AluI for the *htrA* gene in 8 pools of samples. Lanes: ladder 50 bp molecular weight marker (ZYMO RESEARCH); lanes 1–8, samples; lane 9, positive control (*Rickettsia slovaca*). The restriction pattern corresponds to *Rickettsia felis* according to the size of the fragments generated by the digestion (177, 119 and 109 bp).

**Figure 2.**
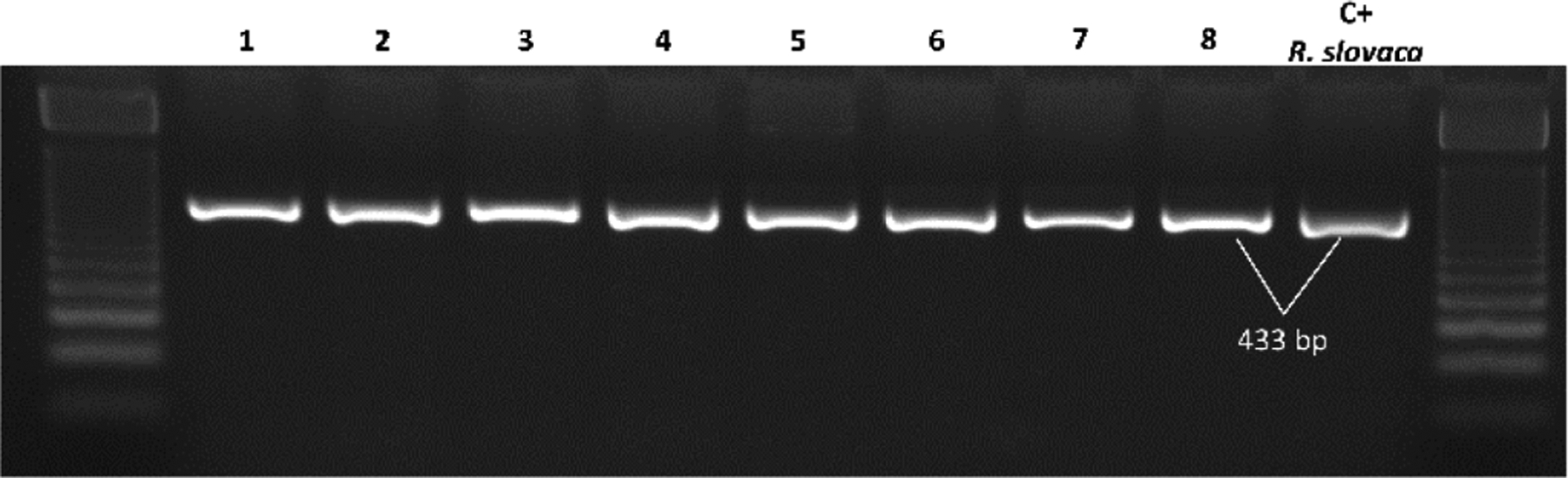
Restriction patterns with XbaI for the *htrA* gene in 8 pools of samples. Lanes: ladder 50 bp molecular weight marker (PROMEGA); lanes 1–8, samples; lane 9, positive control (*Rickettsia slovaca*); ladder 50 bp. *Rickettsia felis* and *R. slovaca* have no restriction site for this enzyme (fragment size: 433 bp).

A sample of 169 of the amplified products for the *htrA* gene, were regrouped into 79 pools according to the municipality of origin, host and flea species. Each pool for RFLP analysis had a maximum of 5 amplified products positive for *htrA* (randomly chosen); and were further divided into two aliquots to be digested with AluI and XbaI. The obtained results were consistent with *R. felis* restriction patterns, previously generated *in silico* for both endonucleases (Figures 1 and 2).

### Rickettsia species identification by sequencing in flea samples

**Figure 3.**
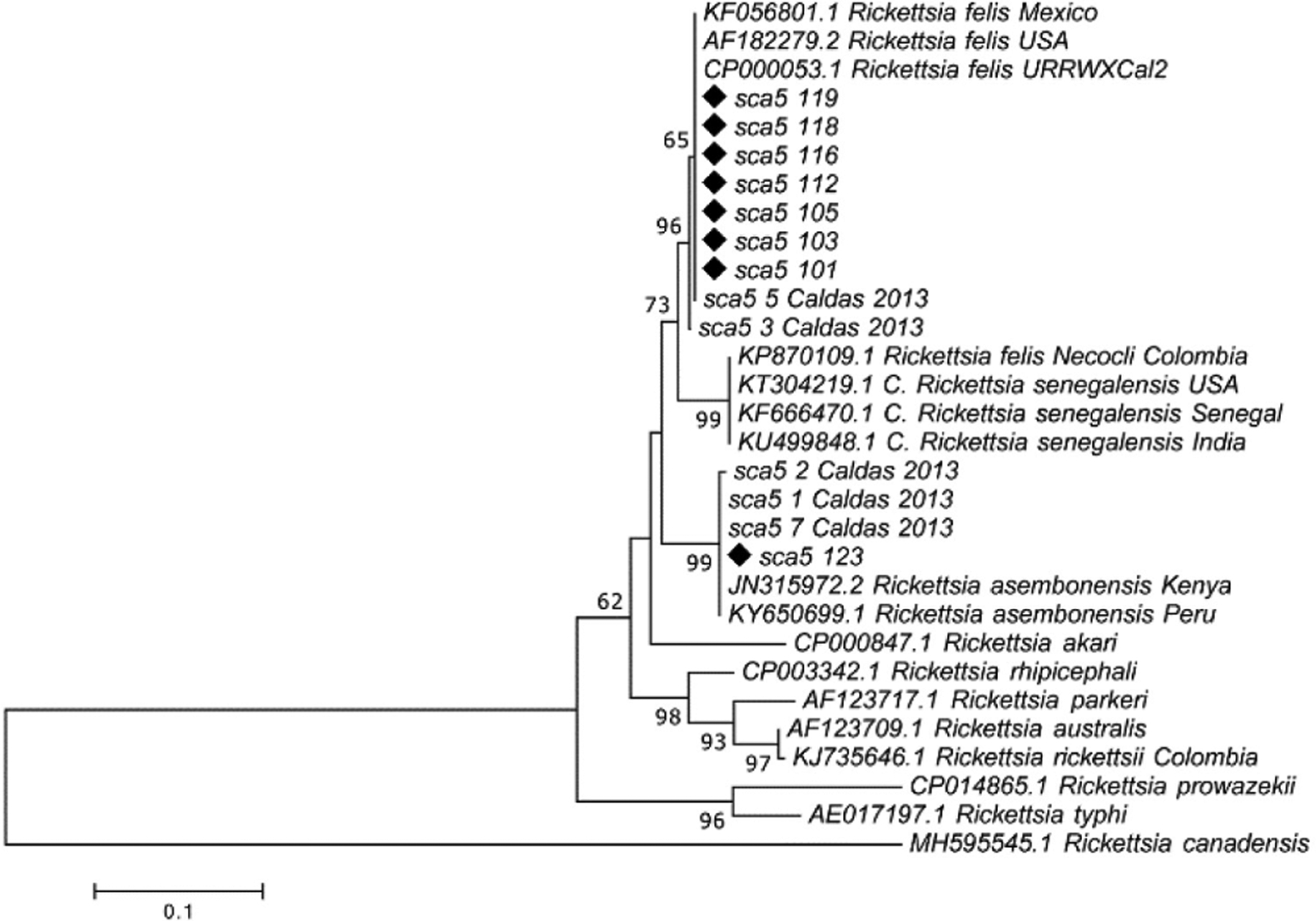
Molecular phylogenetic analysis of *Rickettsia sca5* gene. The evolutionary history was inferred by using the Maximum Likelihood method based on the Tamura-Nei model. The percentage of trees in which the associated taxa clustered together is shown next to the branches. The tree is drawn to scale, with branch lengths measured in the number of substitutions per site. Sequences obtained in the present study are marked with black diamonds. The accession number for each sequence is indicated.

**Figure 4.**
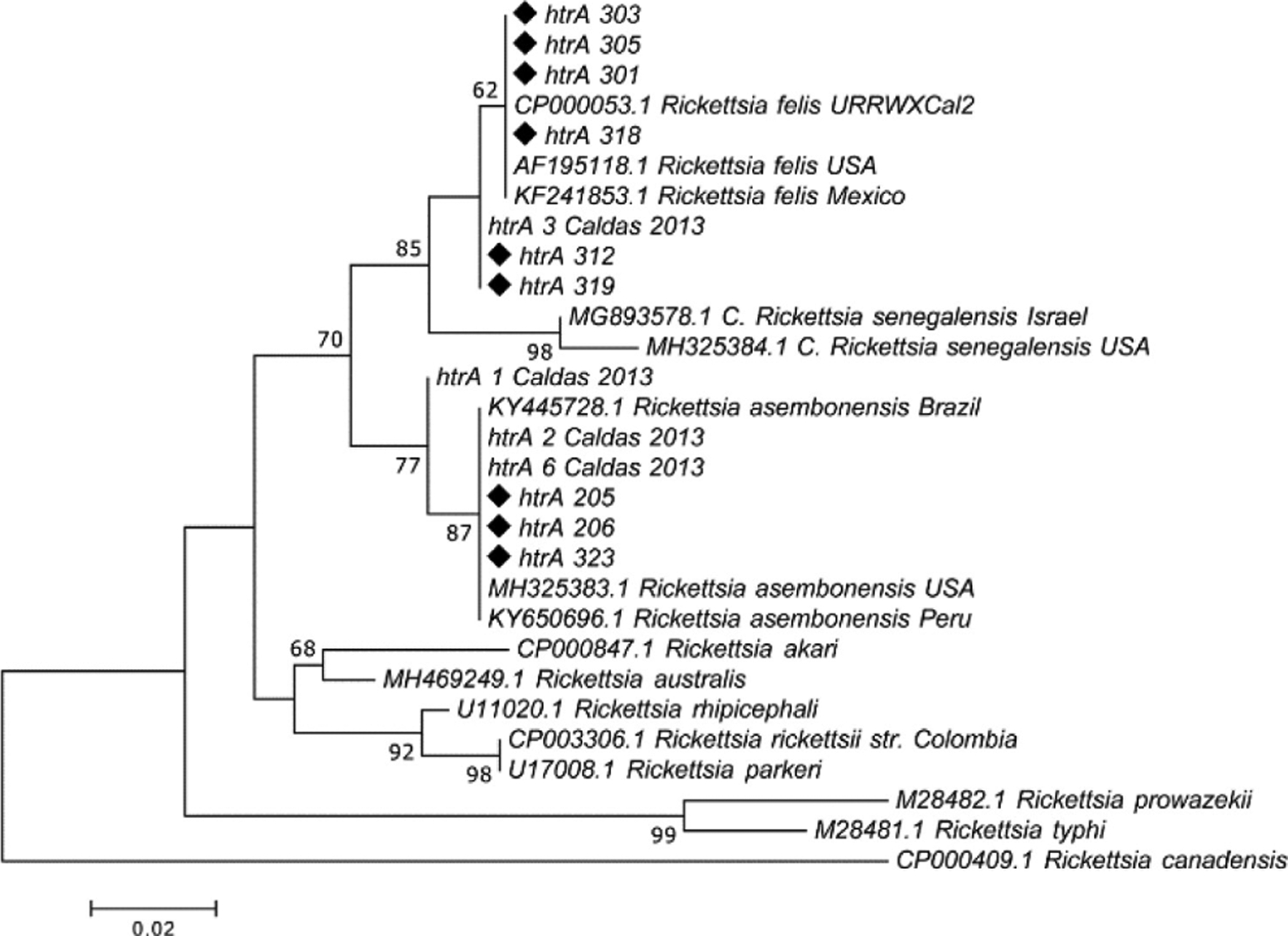
Molecular phylogenetic analysis of *Rickettsia htrA* gene. The evolutionary history was inferred by using the Maximum Likelihood method based on the Tamura-Nei model. The percentage of trees in which the associated taxa clustered together is shown next to the branches. The tree is drawn to scale, with branch lengths measured in the number of substitutions per site. Sequences obtained in the present study are marked with black diamonds. The accession number for each sequence is indicated.

For sequencing, we randomly selected 28 different flea pools as follows: 8 positives for *gltA*, 8 positives for *sca5* and 12 positives for *htrA*, respectively. The criteria for inclusion were based on municipality of origin, host and flea species. All electropherograms obtained were of good quality for editing and alignment analysis. By *gltA* sequence alignment, *R. asembonensis* (1 pool, identity > 99%) and *R. felis* (7 pools, > 98%) were identified; by *sca5* the same species and number of pools were identified (*R*. *asembonensis*, 1, > 99%; *R*. *felis*, 7, > 98%), and, finally, by *htrA* sequence alignment, *R. felis* (8 pools, > 98%) and *R. asembonensis* (4 pools; > 98%) were identified. Phylogenetic trees were constructed with some sequences from *sca5* and *htrA* genes. In the phylogenetic tree constructed for *sca5*, 7 sequences grouped within an *R*. *felis* clade and 1 sequence within an *R*. *asembonensis* clade (Figure 3). Similarly, in the *htrA* tree, 6 sequences grouped within an *R*. *felis* clade and 3 sequences within an *R*. *asembonensis* clade (Figure 4).

## Discussion

*Ctenocephalides felis* was the main flea species collected in the present study, from both domestic animals and wild mammals, demonstrating that it is a multi-host and ubiquitous ectoparasite that can serve as a vector for *Rickettsia*, among other pathogens, and represents a risk of exposure for human populations due to the close contact with domestic and synanthropic hosts [41]. These results are in accordance with a previous study in seven municipalities of Caldas Department, in which *C*. *felis* was the dominant species, collected particularly from dogs and cats [26]. Additionally, in agreement with the latter, *C*. *canis* specimens were the second most prevalent species on domestic animals. In contrast, a smaller number of *P*. *irritans* (35 vs. 4 fleas) and *X*. *cheopis* (16 vs. 8) were obtained. Finally, there was significant diversity of flea species collected from wild mammals. Specimens from the genus *Leptopsylla*, *Nosopsyllus* and *Ctenophthalmus* were identified parasitizing rodents and opossums. Previous reports have detected different pathogenic *Rickettsia* and *Bartonella* species within these fleas in different countries of Africa, Asia and Europe [42–49].

In the present study, Minimum Infection Rates (MIR%) ranged between 10 and 100%, as determined by the three genes amplified, which are higher in comparison to those reported by Ramírez-Hernández *et al*. [26] (2.7–50%) in this department. This variance could be due to the lower number of flea specimens included in each pool in the present study (maximum: 3 fleas/pool) compared with the former (max.: 7 fleas/pool); and also due to the small number of fleas collected in some localities and hosts (i.e. wild mammals). Nonetheless, it confirms high variable infection rates in fleas collected from domestic animals as reported elsewhere [50]. Besides, negative amplification in other flea species (those different from *C*. *felis* and *C*. *canis*) could be related to the small number of specimens collected, variability in PCR sensitivity and consequent reduced likelihood of detection.

PCR-RFLP and sequencing of DNA obtained from flea samples confirmed the presence of *R*. *felis* and *R*. *asembonensis*. The results obtained by PCR-RFLP were validated by performing the *in*-*silico* digestion of the *htrA* gene, obtained from the complete genome published in RefSeq [51], with the two endonucleases, *Alu*I and *Xba*I, used in this study. However, the sizes of the restriction patterns differ in very few base pairs with those obtained for *R. felis* and *R*. *asembonensis*, making them difficult to interpret. The sizes of the restriction fragments obtained are consistent with results obtained using similar methodologies in different studies [52–55]. A study drawback was that different samples for each gene were included in sequencing reactions, which probably reduced species identification accuracy. Nonetheless, we consider that species identification is well supported through the number of sequences obtained and phylogenetic trees constructed.

*Rickettsia felis*, a widely distributed species reported in different arthropods from all continents except Antarctica [41], has been previously detected in Colombia in several flea species from Caldas [26] and Cundinamarca [56]. In the former, sequences were obtained from *C*. *felis*, *C*. *canis* and *P*. *irritans* collected from domestic animals from six municipalities (Aguadas, Aranzazu, Filadelfia, Neira, Pácora and Salamina); and, in the latter, sequences were acquired from a *C*. *felis* flea obtained in a human bed from Villeta. On the other hand, *R*. *asembonensis*, a flea-borne species originally described in fleas collected in 2009 in Asembo (Kenya) [51,57,58], which has also been identified in different South American countries including Brazil [59,60], Ecuador [61] and Peru [62,63], has only been identified in Colombia in *C*. *felis* from Villeta (Cundinamarca) [56]. The pathogenicity of this *R*. *felis*-like species in vertebrates is unknown and must be clarified by further ecologic and experimental studies [2].

The Department of Caldas has been recognized as an endemic area for murine typhus since the first cases were recognized and reported in 1940 [23]. Further studies corroborated the disease in febrile patients [24,25] and the active circulation of flea-borne *Rickettsia* species, with seven municipalities demonstrating a high seroprevalence (71.7%) [24]. Furthermore, a subsequent ecologic study examining fleas detected *R. felis* in the same localities [26]. Although the specific etiologic cause of the febrile syndrome compatible with murine typhus is unknown in this region, the results herein obtained suggest the circulation of flea-borne *Rickettsia* species (e.g., *R. asembonensis* and *R. felis*) in fleas from domestic (i.e. dogs and cats) and wild mammals in a higher number of municipalities than previously recognized. We cannot discard the circulation of *R*. *typhi* in this territory. A small number of rodents captured and a subsequent small number of *X*. *cheopis* collected, which is recognized as the primary vector [64], could explain why it was not detected.

It is worthy to note that presented phylogenetic trees for *sca5* and *htrA* included sequences obtained from a previous study of fleas collected in the Caldas department between 2010 and 2011 [26]. Although in this work they were identified as *R*. *felis*, here, some sequences (i.e. sequences identified as “Caldas 2013” in figures 3 and 4) grouped within the *R*. *asembonensis* clade (3 and 2 sequences with *sca5* and *htrA*, respectively). Those results, and those obtained with flea samples from the present study, corroborate that both *Rickettsia* species have been circulating in Caldas Department since, at least, 2010.

## Conclusion

In conclusion, we found two flea-borne rickettsiae (i.e., *R*. *felis* and *R*. *asembonensis*) in fleas from pets and synanthropic animals in close contact with the human population. Even though many epidemiological, ecological and pathogenic questions must be resolved, healthcare providers should be aware of flea-borne rickettsioses as a potential diagnosis in patients with acute febrile illness.

## Supplementary Material

1

## Figures and Tables

**Table 1.** Flea species collected in domestic animals and wild mammals between November 2015 and January 2017, in 26 municipalities from Caldas Department, Colombia.

	Flea species						
Municipality	*Ctenocephalides felis*			*Ctenocephalides canis*			Other flea species^b^
	Dog^a^	Cat	WM	Dog	Cat	WM	
Aguadas	-	-	-	-	-	-	
Anserma	-	43 (3.9)	-	4 (1.6)	-	-	
Belalcazar	29 (2.6)	32 (2.9)	-	-	2 (0.8)	-	*Pulex irritans* (Dog) (2)
Chinchiná	8 (0.7)	43 (3.9)	-	-	1 (0.4)	-	*Xenopsylla cheopis (Didelphis marsupialis)* (1)
Filadelfia	5 (0.5)	-	-	-	-	-	
La Dorada	-	10 (0.9)	-	-	-	-	
La Merced	-	-	-	-	-	-	
Manizales	29 (2.6)	40 (3.6)	-	3 (1.2)	8 (3.1.)	-	*Leptopsylla segnis (Thomasomys* cf. *baeops)* (1), *Leptopsylla* sp. (*Thomasomys* cf. *baeops*) (2), *Ctenophtalmus* sp. (*Nephelomys childi, Thomasomys* cf. *baeops*) (4), *Nosopsyllus* sp. (*Thomasomys* cf. *baeops*) (2)
Manzanares	56 (5.1)	106 (9.7)	-	6 (2.3)	2 (0.8)	-	
Marmato	23 (2.1)	-	10 (0.9)	16 (6.2)	-	3 (1.2)	*Leptopsylla segnis (Didelphis marsupialis)* (1); X. *cheopis (Didelphis marsupialis)* (3)
Marquetalia	20 (1.8)	104 (9.5)	-	4 (1.6)	2 (0.8)	-	
Marulanda	4 (0.4)	18 (1.6)	-	7 (2.7)	2 (0.8)	-	
Neira	-	5 (0.5)	-	-	-	-	
Norcasia	1 (0.1)	40 (3.6)	-	42 (16.3)	-	-	
Pácora	-	-	-	-	-	-	*Leptopsylla segnis* (*Didelphis marsupialis*) (6); *X*. *cheopis* (*Didelphis marsupialis*) (1)
Palestina	45 (4.1)	23 (2.1)	-	-	-	-	*Rhopalopsyllus* spp. (*Didelphis marsupialis*) (1)
Pensilvania	6 (0.6)	21 (1.9)	-	-	-	-	
Riosucio	2 (0.2)	46 (4.2)	-	-	-	-	P. *irritans* (Cat) (2)
Risaralda	25 (2.3)	-	-	23 (8.9)	-	-	
Salamina	-	-	-	-	-	-	
Samaná	18 (1.6)	14 (1.3)	-	-	-	-	
San José	28 (2.6)	42 (3.8)	-	-	-	-	
Supía	1 (0.1)	44 (4.0)	-	40 (15.5)	-	-	*X*. *cheopis* (Cat) (1)
Victoria	14 (1.3)	36 (3.3)	-	62 (24)	-	-	*X*. *cheopis* (*Proechymis* spp.) (2)
Villamaria	15 (1.4)	17 (1.6)	-	24 (9.3)	3 (1.2)	-	*Leptopsylla segnis* (*Nephelomys childi*, *Riphidomys cf. latimanus*) (2)
Viterbo	20 (1.8)	56 (5.1)	-	-	-	-	
Total	349 (31.8)	740 (67.3)	10 (0.9)	235 (91.1)	20 (7.8)	3 (1.2)	31 (0.02)
		1099 (79.4)			258 (18.6)		
							1388

WM: Wild mammals;

a. Data are presented as No. of positive pools/Total number of fleas tested (Minimum Infection Rate-MIR);

b. Municipalities without flea samples (i.e. Aguadas, La Merced and Salamina) were not included.

**Table 2.** Flea samples collected between November 2015 and January 2017, in different municipalities from Caldas Department (Colombia), positive for *Rickettsia* DNA by gene and host.

	Flea samples^a^								
Municipality^b^	*gltA*			*sca5*			*htrA*		
	Dogs	Cats	WM	Dogs	Cats	WM	Dogs	Cats	WM
Anserma	-	30/47 (0.6)	-	-	29/47 (0.6)	-	-	28/47 (0.6)	-
Belalcazar	11/31 (0.4)	25/34 (0.7)	-	15/31 (0.5)	3/34 (0.1)	-	16/31 (0.5)	28/34 (0.8)	-
Chinchiná	2/12 (0.2)	28/44 (0.6)	1/1 (1.0)	5/12 (0.4)	27/44 (0.6)	0/1 (0)	6/12 (0.5)	29/44 (0.7)	1/1 (1.0)
Filadelfia	3/5 (0.6)	-	-	3/5 (0.6)	-	-	3/5 (0.6)	-	-
La Dorada	-	4/10 (0.4)	-	-	0/10 (0)	-	-	4/10 (0.4)	-
Manizales	21/32 (0.7)	21/48 (0.4)	0/6 (0)	19/32 (0.6)	12/48 (0.3)	6/6 (1.0)	22/32 (0.7)	29/48 (0.6)	0/6 (0)
Manzanares	28/62 (0.5)	54/108 (0.5)	-	17/62 (0.3)	50/108 (0.5)	-	31/62 (0.5)	58/108 (0.5)	-
Marmato	30/39 (0.8)	-	9/17 (0.5)	10/39 (0.3)	-	12/17 (0.7)	30/39 (0.8)	-	17/17 (1.0)
Marquetalia	12/24 (0.5)	54/106 (0.5)	-	8/24 (0.3)	34/106 (0.3)	-	12/24 (0.5)	58/106 (0.6)	-
Marulanda	8/11 (0.7)	8/20 (0.4)	-	8/11 (0.7)	8/20 (0.4)	-	9/11 (0.8)	8/20 (0.4)	-
Neira	-	3/5 (0.6)	-	-	1/5 (0.2)	-	-	3/5 (0.6)	-
Norcasia	30/43 (0.7)	29/40 (0.7)	-	-	11/40 (0.3)	-	30/43 (0.7)	29/40 (0.7)	-
Pácora	-	-	0/7 (0)	-	-	1/7 (0.1)	-	-	3/7 (0.4)
Palestina	30/45 (0.7)	18/23 (0.8)	0/1 (0)	-	-	1/1 (1.0)	30/45 (0.7)	18/23 (0.8)	0/1 (0)
Pensilvania	6/6 (1.0)	13/21 (0.6)	-	-	0/21 (0.0)	-	6/6 (1.0)	14/21 (0.7)	-
Riosucio	1/2 (0.5)	28/48 (0.6)	-	1/2 (0.5)	28/48 (0.6)	-	1/2 (0.5)	28/48 (0.6)	-
Risaralda	42/48 (0.9)	-	-	-	-	-	42/48 (0.9)	-	-
Samaná	18/18 (1.0)	11/14 (0.8)	-	-	11/14 (0.8)	-	18/18 (1.0)	11/14 (0.8)	-
San José	14/28 (0.5)	26/42 (0.6)	-	14/28 (0.5)	25/42 (0.6)	-	14/28 (0.5)	26/42 (0.6)	-
Supía	30/41 (0.7)	31/45 (0.7)	-	-	-	-	30/41 (0.7)	31/45 (0.7)	-
Victoria	30/76 (0.4)	30/36 (0.8)	0/2 (0)	-	-	0/2 (0)	30/76 (0.4)	30/36 (0.8)	2/2 (1.0)
Villamaría	29/39 (0.7)	1/20 (0.1)	0/2 (0)	12/39 (0.3)	6/20 (0.3)	0/2 (0)	29/39 (0.7)	15/20 (0.8)	2/2 (1.0)
Viterbo	15/20 (0.8)	34/56 (0.6)	-	14/20 (0.7)	33/56 (0.6)	-	15/20 (0.8)	37/56 (0.7)	-
Total	360/582 (0.6)	448/767 (0.6)	10/36 (0.3)	126/305 (0.4)	278/663 (0.4)	20/36 (0.6)	374/582 (0.6)	484/767 (0.6)	25/36 (0.7)

WM: Wild mammals;

a. Data are presented as No. of positive pools/Total number of fleas tested (Minimum Infection Rate-MIR);

b. Municipalities without flea samples (i.e. Aguadas, La Merced and Salamina) were not included.
