# Supplementary material for: Flea-borne Rickettsia species in fleas, Caldas department, Colombia
Source: J Infect Dev Ctries. Author manuscript; Available in PMC 2025 Feb 13. (PMC11823469; doi:10.3855/jidc.12524)
Supplement: 1 [file NIHMS2052927-supplement-1.pdf]

Annex – Supplementary Items

Supplementary Table 1. Geographical coordinates of municipalities included in the study.

Municipality	Geographical coordinates	
	Latitude	Longitude
Aguadas	5.612458	-75.457548
Anserma	5.232087	-75.78527
Belalcazar	4.994312	-75.812254
Chichiná	4.984308	-75.604848
Filadelfia	5.298106	-75.560822
La Dorada	5.453972	-74.667146
La Merced	5.399049	-75.546934
Manizales	5.06768	-75.509819
Manzanares	5.253492	-75.153634
Marmato	5.474164	-75.598926
Marquetalia	5.296501	-75.053844
Marulanda	5.284023	-75.259838
Neira	5.165727	-75.520327
Norcasia	5.574879	-74.888868
Pácora	5.526423	-75.459719
Palestina	5.020476	-75.623254
Pensilvania	5.383393	-75.16118
Riosucio	5.420838	-75.703171
Risaralda	5.167189	-75.76587
Salamina	5.406654	-75.487369
Samaná	5.412465	-74.99264
San José	5.081215	-75.791367
Supía	5.451751	-75.651113
Victoria	5.317354	-74.912025
Villamaría	5.044416	-75.51431
Viterbo	5.062456	-75.87224

Supplementary Figure 1. Localization of Caldas Department and its municipalities. Aranzazu (no. 3 in the map) was not included in the study.

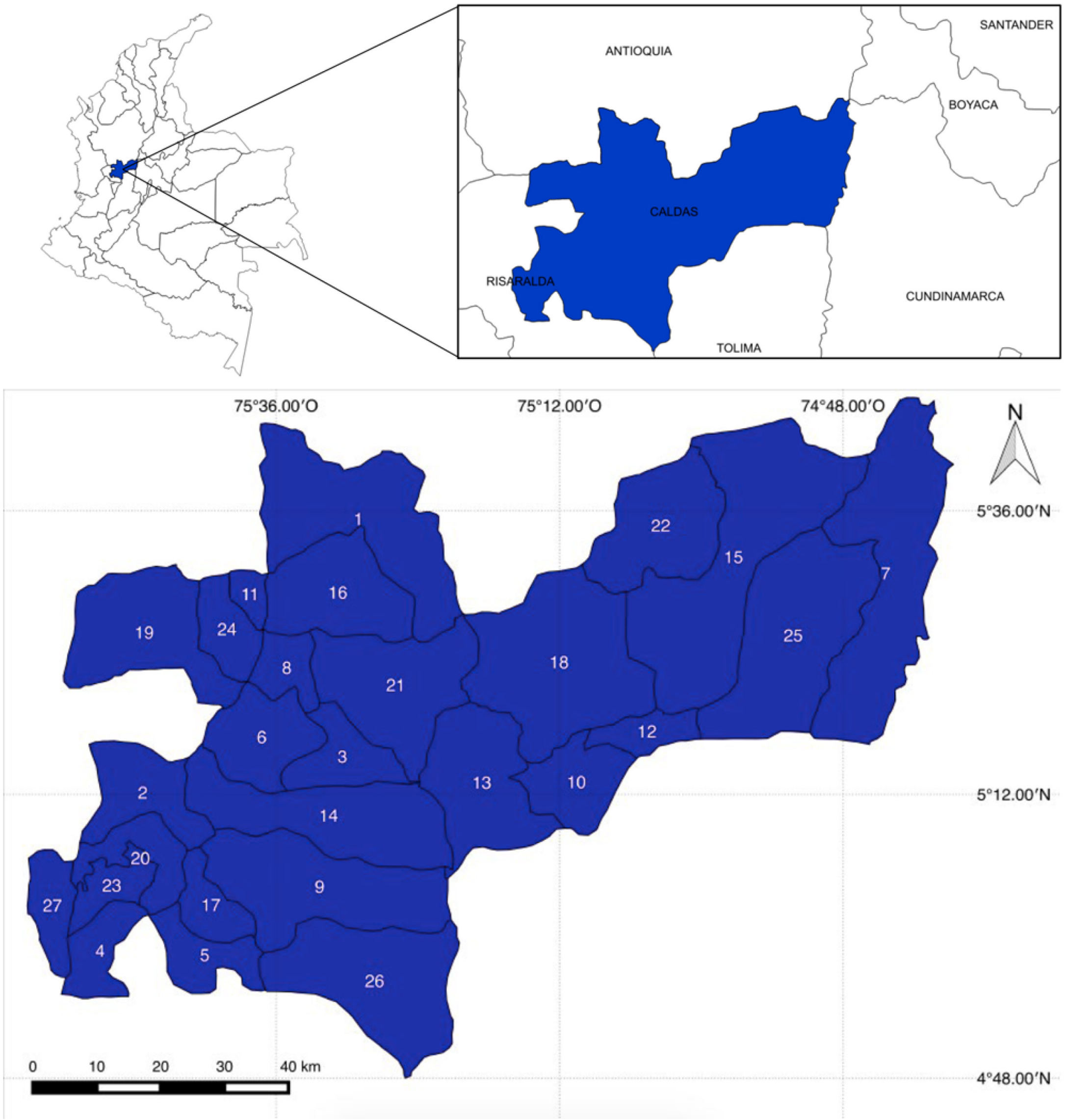

No.	Municipality
1	Aguadas
2	Anserma
3	Aranzazu
4	Belalcazar
5	Chichiná
6	Filadelfia
7	La Dorada
8	La Merced
9	Manizales
10	Manzanares
11	Marmato
12	Marquetalia
13	Marulanda
14	Neira
15	Norcasia
16	Pácora
17	Palestina
18	Pensilvania
19	Riosucio
20	Risaralda
21	Salamina
22	Samaná
23	San José
24	Supía
25	Victoria
26	Villamaría
27	Viterbo
